# RECRUITING OLDER ADULTS WITH DEMENTIA FOR NEUROIMAGING RESEARCH: BARRIERS AND SOLUTIONS

**DOI:** 10.1093/geroni/igz038.2201

**Published:** 2019-11-08

**Authors:** Sebastian Atalla, Kathy Wright, Alison Anderson, Karen O Moss, Ronald Cowan, Ann McDaniel, Todd Monroe

**Affiliations:** 1 The Ohio State University, columbus, Ohio, United States; 2 Vanderbilt University, Nashville, Tennessee, United States

## Abstract

Recruiting participants with dementia for magnetic resonance imaging (MRI) research is prone to inherent barriers including caregiver schedules, mistrust, transportation, expertise of MRI technologists, claustrophobia, and MRI safety requirements. This pilot study aimed to identify barriers and develop strategies for increasing recruitment and retention of persons with dementia in neuroimaging studies and to gauge improvements in recruitment outcomes associated with dedicated recruitment staff. Over a period of five years of active recruitment, a dedicated recruitment specialist and a full-time research assistant screened and enrolled an average of eight participants with dementia per month (8.16), of which, approximately two participants (1.97) successfully completed the MRI testing. The most common barrier was difficulty obtaining surgical records for MRI safety clearance. The most common solutions were thorough prescreening and maintaining a positive rapport with MRI technologists. Our study findings will assist others with engaging persons with dementia and their caregivers in MRI research.

